# Adiposity cut-off points for cardiovascular disease and diabetes risk in the Portuguese population: The PORMETS study

**DOI:** 10.1371/journal.pone.0191641

**Published:** 2018-01-29

**Authors:** Luís Raposo, Milton Severo, Ana Cristina Santos

**Affiliations:** 1 Insulin Resistance Study Group of the Portuguese Society of Endocrinology, Diabetes and Metabolism, Lisboa, Portugal; 2 EPIUnit—Instituto de Saúde Pública, Universidade do Porto, Porto, Portugal; 3 Departamento de Ciências da Saúde Pública e Forenses e Educação Médica, Faculdade de Medicina, Universidade do Porto, Porto, Portugal; Universitat de les Illes Balears, SPAIN

## Abstract

**Objectives:**

The contribution of adiposity to cardiovascular and diabetes risk justifies the inclusion of an adiposity measure, usually waist circumference, in the definition of metabolic syndrome. However, waist circumference thresholds differ across populations. Our aim was to assess which adiposity measure performs the best in identifying the metabolic syndrome in a sample of Portuguese participants and to estimate cut-off values for these measures.

**Methods:**

Data were obtained from a cross-sectional study (PORMETS study) conducted in Portugal between 2007 and 2009. A representative sample of non-institutionalized adults, comprising 3,956 participants, aged 18 years and older, was evaluated. A structured questionnaire was administered, collecting information on personal medical history, socio-demographics and behavioral characteristics. Anthropometrics, blood pressure and venous blood samples were also obtained. Metabolic syndrome was defined according to the Joint Interim Statement of the International Diabetes Federation Task Force on Epidemiology recommended criteria. Elevated cardiometabolic risk was considered when two or more of the four criteria of metabolic syndrome were present, excluding the waist circumference component. A receiver operating characteristic curve was used to estimate cut-off points.

**Results:**

This study found that waist-to-height ratio, waist circumference and body adiposity index performed better than other adiposity measures, such as body mass index. The estimated cut-off points for waist-to-height ratio, waist circumference and body adiposity index in women and men were 0.564 / 89 cm / 27.4 and 0.571 / 93.5 cm / 25.5, respectively.

**Conclusion:**

As waist circumference is currently used as the adiposity measure in the definition of metabolic syndrome and as no relevant differences were observed between this measure and waist-to-height ratio, it is likely that no modification to the metabolic syndrome definition needs to be proposed. Moreover, this study also confirmed the applicability of European cut-off points in the Portuguese population.

## Introduction

The prevalence of obesity in Portugal [1] and worldwide [2] has risen to “pandemic” proportions. Obesity is independently related to type 2 diabetes [3], coronary heart disease, cerebrovascular disease and increased all-cause mortality as well as mortality after cardiovascular events [4, 5]. Despite the established contribution of overall and abdominal obesity to the cardiometabolic risk, visceral adipose tissue (VAT) may have a stronger impact than total body fat (TBF) on insulin resistance, beta cell dysfunction and atherosclerosis [6].

TBF can be estimated by weight (Wt), and other adiposity indicators that are often indexed to height (Ht) and include body mass index (BMI) and body adiposity index (BAI) [7]. Despite its good correlation with TBF, BMI is only moderately correlated with VAT [8].

To assess the distribution of adiposity, other indicators are available and calculated from anthropometric parameters [9], such as waist circumference (WC), hip circumference (HC) and several ratios, including waist-to-hip ratio (WHR) and waist-to-height ratio (WHtR). When compared to BMI, WC and WHtR in both sexes and WHR in men presented stronger associations with VAT [10].

The superiority of measures of abdominal obesity (AO), namely WC and WHtR, over BMI for predicting cardiovascular disease (CVD) [11] and diabetes risk [3] has been increasingly documented, and a recent meta-analysis [12] even suggested that WHtR was modestly superior to WC for CVD risk assessment.

The contribution of obesity to CVD and diabetes risk justifies the inclusion of an adiposity measure, usually WC, in the most frequently used definitions of metabolic syndrome (MetS) [13]. MetS includes a group of interconnected clinical and metabolic factors that increases the risk of developing CVD in 5 to 10 years [14] and confers a 3- to 5-fold increased risk of type 2 diabetes [15]. These factors include dysglycemia, increased blood pressure, elevated triglyceride levels, low high-density lipoprotein cholesterol (HDL-C) levels, and AO.

The two most frequently used gender-specific thresholds for WC in the European population resulted from a proposal of the National Institutes of Health [16] based on a study in Caucasian populations [17]; this proposal established a BMI of ≥25 kg/m^2^ or ≥30 kg/m^2^ to correspond to WC cut-points of 80 or 88 cm for women and of 94 or 102 cm for men, respectively. However, an increasing number of published papers have documented a variation in the optimal cut-off values for WC in different populations [18, 19]. Therefore, the same methodology used to establish WC cut-off points may be applied to other adiposity measures.

The primary objective of the present study was to assess which adiposity measure performs the best in identifying MetS in a sample of Portuguese adults. The secondary objective was to estimate the cut-off values for these adiposity measures in the same sample.

## Participants and methods

**Fig 1:**
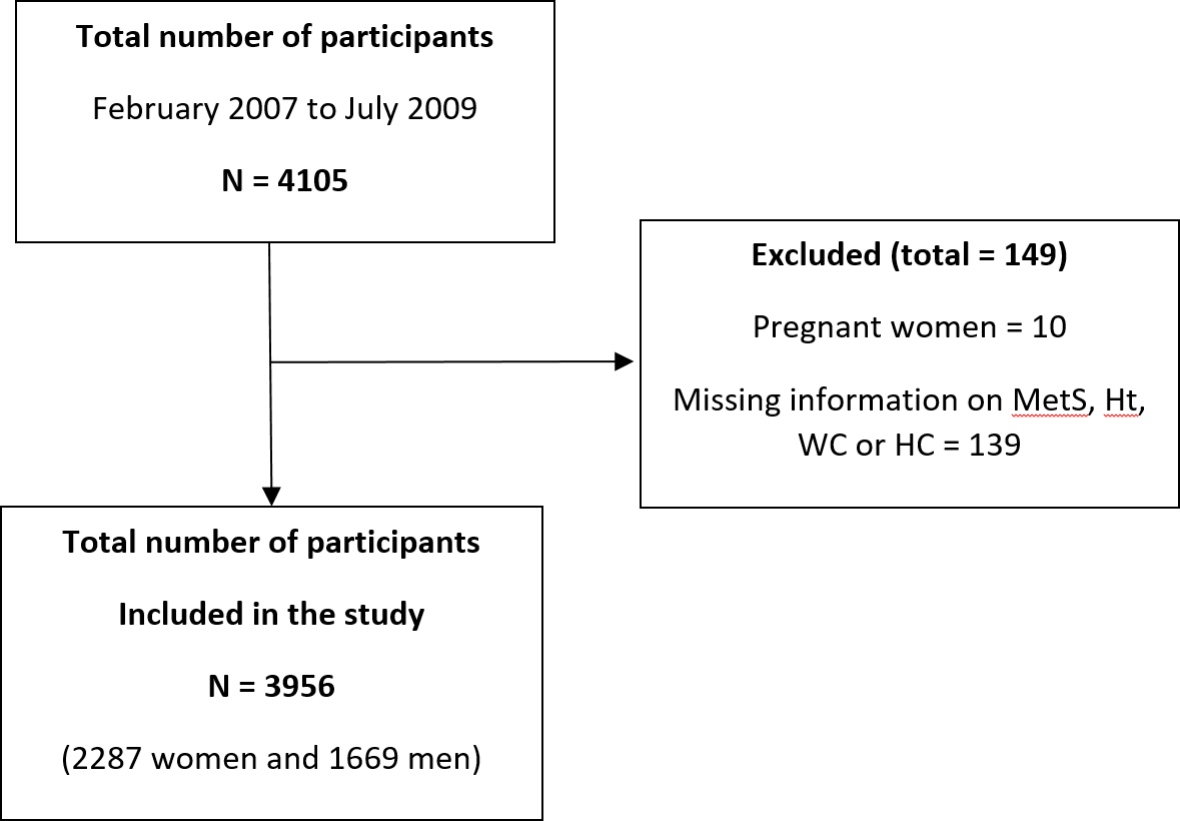
Flow chart of the PORMETS study. MetS, metabolic syndrome; Ht, height; Wt, weight; WC, waist circumference; HC, hip circumference.

PORMETS is a cross-sectional study comprising a representative sample of adults registered at primary health care centers in mainland Portugal [20]. A cluster sample, representative of the Portuguese population, was obtained after stratification by selected districts and urban/rural areas. A total of 4,105 participants, aged 18 years and older, were evaluated, and the information was collected from February 2007 to July 2009. Ten women were excluded from the data analysis because they were pregnant at the time of the interview, resulting in 4,095 participants. After excluding participants with missing information on MetS, Ht, Wt, WC, and HC, the remaining 3,956 participants (2,287 women and 1,669 men) were included in the final data analysis (Fig 1).

PORMETS was approved by all Portuguese Regional Health Administrations, by the Ethics Committee of Centro Hospitalar São João (authorized in 27^th^ February 2007) and by the Portuguese Data Protection Authority (authorization number: CNPD 1053/2007). Additionally, authorization was provided by the Clinical Director of each health care center. All participants provided written informed consent.

A structured questionnaire was administered by trained nurses collecting information on personal medical history and socio-demographic and behavioral characteristics, such as smoking, intake of alcoholic beverages and engagement in physical exercise.

Participants were considered current smokers if they smoked daily or occasionally, former smokers if they had stopped smoking for at least 6 months, and non-smokers if they had never smoked. Regarding alcohol intake, participants were categorized as occasional drinkers if they had less than one drink per day, daily drinkers if they consumed at least one drink per day, former drinkers if they had stopped drinking for at least 6 months and non-drinkers if they had never consumed any type of alcoholic beverage.

Adiposity measurements were collected, namely Wt, WC and HC. Body Wt was measured to 0.1 kg using a digital scale, and Ht to the nearest centimeter using a wall stadiometer with participants in the standing position. WC was measured midway between the bottom of the rib cage and the iliac crest, and HC was measured as the maximum circumference of the buttocks. BMI was calculated as Wt in kilograms divided by the square of Ht in meters. Participants were classified according to the World Health Organization criteria: overweight was defined as BMI ≥ 25 to < 30 kg/m^2^ and obesity as BMI ≥ 30 kg/m^2^. The WHtR was calculated as the WC divided by the Ht, and the WHR, as the WC divided by the HC, all in centimeters. BAI [8] was calculated using the formula BAI = [(HC cm) / (Ht m)^1.5^)]– 18.

Blood pressure was measured on a single occasion, using a standard mercury sphygmomanometer with the cuff on the right upper arm, after a 10-minute rest. Two blood pressure readings were obtained, and the mean of the two readings was registered. When the difference between the two measurements was larger than 5 mm Hg for systolic or diastolic blood pressure, a third measurement was taken and the mean of the two closest values was used.

A fasting venous blood sample was collected by trained nurses in each health care center. All blood sampling analyses were performed centrally at the Department of Clinical Pathology, Centro Hospitalar São João, Porto, Portugal. Glucose, total cholesterol, HDL-C and triglycerides were determined using automatic standardized routine enzymatic methods. High sensitivity C-reactive protein (hs-CRP) levels were determined by particle-enhanced immunonephelometry. Insulin was measured using a ^125^I-labelled insulin radioimmunoassay method, and insulin resistance was estimated by the homeostatic model assessment (HOMA-IR) as the product of fasting glucose (mmol/L) and insulin (μUI/mL) divided by a constant 22.5 [21].

MetS was defined according to the Joint Interim Statement of the International Diabetes Federation Task Force on Epidemiology (JIS) recommended criteria [13]. MetS was considered present if at least three of the five following criteria were identified: fasting glucose ≥ 100 mg/dL (5.6 mmol/L) or antidiabetic treatment; blood pressure ≥130/85 mmHg or antihypertensive medication; triglycerides ≥150 mg/dL (1.7 mmol/L) or specific lipid-lowering therapy; HDL-C <50 mg/dL (1.29 mmol/L) in women and <40 mg/dL (1.03 mmol/L) in men or specific treatment for this lipid abnormality; and, using 2 cut-off points according to the European Cardiovascular Societies (*European*) or the International diabetes Federation (IDF) criteria (*Europid*), a WC greater than or equal to 102 or 94 cm in males and 88 or 80 cm in females, respectively.

Elevated cardiometabolic risk was considered present when two or more of the four JIS criteria were present, excluding the WC component [10, 22].

### Statistical analysis

The association between the adiposity measures and adverse health outcomes (dichotomous variable) was estimated by point-biserial correlation.

Receiver operating characteristic (ROC) curves were created to compare the ability of several adiposity measures (Wt, BMI, HC, WC, WHtR, WHR and BAI) to identify elevated cardiometabolic risk and to estimate the cut-off points that better identified adverse health outcomes. The area under the ROC curve (AUC) was calculated for each adiposity measure, and the cut-off points were estimated using the point that maximized the sensitivity plus specificity. The application of this method was supported by its preferential use, as demonstrated in previous studies [18, 19]. The 95% confidence intervals (95% CI) for cut-off points were estimated using percentile bootstrap confidence interval calculations, based on 200 bootstrap replicate.

Sensitivity, specificity, positive and negative predictive values, accuracy, and positive and negative likelihood ratios (LR) were calculated for each estimated cut-off point.

Continuous variables were described as the means and standard deviations (SD) or as the median and corresponding 25^th^ and 75^th^ percentiles for non-normally distributed variables. Counts and proportions were reported for categorical variables. Proportions were compared using a chi-square test or Fisher’s exact test, as appropriate. T-tests for two independent samples or Mann-Whitney test were also used to compare continuous variables. A p value < 0.05 was considered statistically significant. All statistical analyses were performed using SPSS® version 21 (IBM, Armonk, New York, US).

## Results

This study comprised 3,956 individuals, of whom 2,287 were women (57.8%). The mean (±SD) age in this sample was 53.2 (±16.3) years; women were 52.6 (±16.3) years old on average, and men were 54.1 (±16.4) years old. In general, women had lower systolic and diastolic blood pressure (p<0.001), fasting glucose (p<0.001) and triglycerides (p<0.001) but higher HDL-C (p<0.001), insulin (p = 0.002) and hs-CRP (p<0.001) levels than men (Table 1).

**Table 1:** Sample demographic, behavioral and analytical characteristics according to gender.

	Women	Men	p-value
**Overall [N (%)]**	2287 (57.8)	1669 (42.4)	
**Age (years)–mean (SD)**	52.6 (16.3)	54.1 (16.4)	0.004
**Education (years)–mean (SD)**	6.7 (4.7)	6.8 (4.3)	0.562
**Regular physical exercise [N (%)]**	561 (24.5)	495 (29.7)	<0.001
**Smoking**			
**Never smoker [N (%)]**	1934 (85.5)	822 (50.3)	
**Former smoker [N (%)]**	116 (5.1)	452 (27.6)	
**Current smoker [N (%)]**	212 (9.4)	361 (22.1)	<0.001
**Alcohol intake**			
**Non-drinker [N (%)]**	1501 (66.1)	284 (17.1)	
**Former drinker [N (%)]**	55 (2.4)	81 (4.9)	
**Occasional drinker [N (%)**	517 (22.8)	515 (31.0)	
**Daily drinker [N (%)]**	199 (8.8)	779 (47.0)	<0.001
**Systolic BP (mmHg)—Mean (SD)**	129.0 (22.2)	135.9 (21.8)	<0.001
**Diastolic BP (mmHg)—Mean (SD)**	77.2 (12.1)	79.8 (12.1)	<0.001
**Total cholesterol (mg/dL)—Mean (SD)**	209.7 (39.7)	207.5 (44.8)	0.114
**Triglycerides (mg/dL)—Mean (SD)**	115.3 (58.6)	134.8 (85.0)	<0.001
**HDL-C (mg/dL)—Mean (SD)**	50.9 (11.8)	43.8 (12.3)	<0.001
**Fasting glucose (mg/dL)—Mean (SD)**	88.72 (25.50)	96.49 (30.60)	<0.001
**Insulin (μIU/mL)–median (P25, P75)**	7.8 (5.3, 11.5)	7.4 (4.7, 11.6)	0.002
**HOMA-IR–median (P25, P75)**	1.64 (1.07, 2.53)	1.66 (1.01, 2.74)	0.694
**hs-CRP (mg/L)–median (P25, P75)**	0.19 (0.08, 0.44)	0.13 (0.07, 0.27)	<0.001

SD, standard deviation; BP, blood pressure; HOMA-IR, homeostatic model assessment-insulin resistance; hs-CRP, high sensitivity C-reactive protein.

For all anthropometric measures there were significant differences between genders (Table 2). Women showed higher values than men for BMI (p = 0.003), HC (p<0.001), WHtR (p = 0.003) and BAI (p<0.001), but lower values of WC (p<0.001) and WHR (p<0.001).

**Table 2:** Adiposity measures and frequency of metabolic syndrome and its individual components according to gender.

	Women	Men	p-value
**Mean (SD)**			
**Weight (kg)**	68.0 (12.8)	78.1 (13.0)	<0.001
**Ht (cm)**	157.2 (6.7)	169.7 (7.2)	<0.001
**WC (cm)**	91.0 (12.5)	97.0 (11.4)	<0.001
**HC (cm)**	104.5 (10.5)	102.3 (8.3)	<0.001
**WHtR**	0.580 (0.085)	0.572 (0.070)	0.003
**WHR**	0.870 (0.078)	0.947 (0.072)	<0.001
**BAI**	28.3 (7.2)	26.0 (5.7)	<0.001
**BMI (kg/m**^**2**^**)**	27.6 (5.1)	27.1 (4.0)	0.003
**N (%)**			
**Overweight (≥ 25 BMI < 30)**	864 (38.4)	773 (47.0)	<0.001
**Obesity (BMI ≥ 30)**	619 (27.5)	373 (22.7)	0.001
**Elevated cardiometabolic risk** **Blood pressure MetS component** **HDL-C MetS component** **Triglycerides MetS component** **Fasting glucose MetS component**	1096 (48.3) 1305 (57.1) 1345 (58.9) 468 (20.5) 417 (18.3)	977 (58.9) 1145 (68.6) 838 (50.2) 513 (30.8) 507 (30.5)	<0.001 <0.001 <0.001 <0.001 <0.001

SD, standard deviation; Ht, height; WC, waist circumference; HC, hip circumference; WHtR, waist-to-height ratio; WHR, waist-to-hip ratio; BAI, body adiposity index; BMI, body mass index; MetS, metabolic syndrome (according to the JIS definition).

The prevalence of elevated cardiometabolic risk was 52.8% (48.3% in women and 58.9% in men) and the prevalence of its components was 62.0%, 55.2%, 24.8% and 23.5% for blood pressure, triglycerides, fasting glucose and HDL-C, respectively. Overall, men presented a higher prevalence of all outcome components (p<0.001) with the exception of HDL-C.

**Fig 2:**
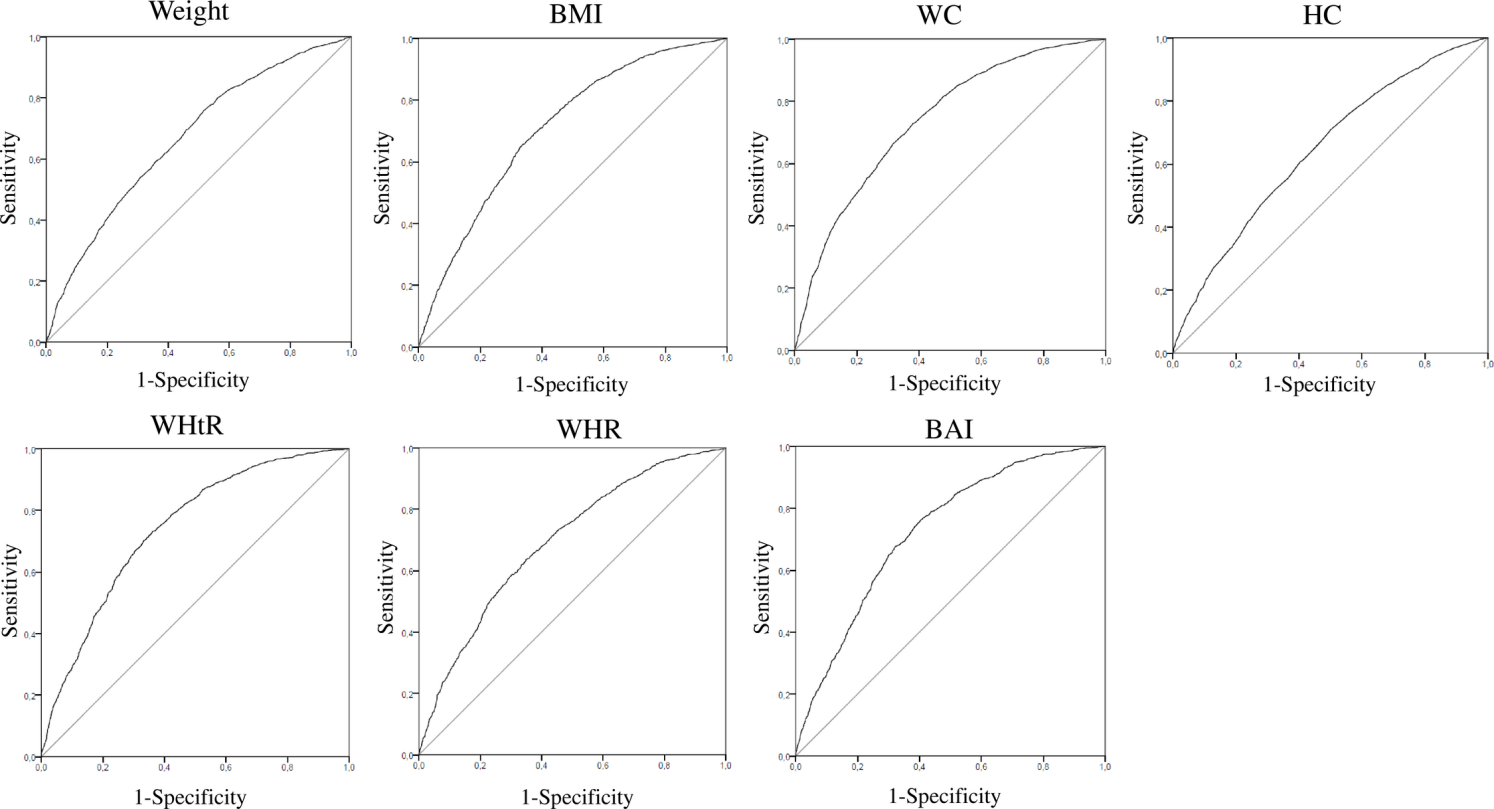
ROC curves of several adiposity measures in terms of the elevated cardiometabolic risk outcome. WHR: waist-to-hip ratio; BMI: body mass index; WC: waist circumference; BAI: body adiposity index; WHtR: waist-to-height ratio.

Table 3 shows the correlations (95% CI), the cut-off points (95% CI), the AUC (95% CI), the performance parameters and the MetS and adiposity component prevalence for all the evaluated adiposity measures. The ability of the adiposity measures to identify elevated cardiometabolic risk, based on the AUC are presented in Fig 2.

**Table 3:** Adiposity measures, elevated cardiometabolic risk and cut-off points performance.

	Elevated CMR correlations (95% CI) ^a^	AUC (95% CI)	Cut-off points (95% CI)	Sn (%)	Sp (%)	PPV (%)	NPV (%)	Accuracy (%)	LR +	LR -	Adipose Comp. (%)	MetS (%)
**Weight(kg) W**	0.276 (0.237, 0.313)	0.664 (0.642, 0.686)	65.0 (63.1, 66.2)	69.4	56.5	59.9	66.4	62.8	1.595	0.542	56.0	38.1
**M**	0.239 (0.196, 0.285)	0.648 (0.621, 0.674)	77.0 (73.2, 79.4)	60.4	62.2	69.6	52.3	61.1	1.598	0.637	51.2	44.0
**BMI(kg/m**^**2**^**) W**	0.352 (0.317, 0.387)	0.714 (0.693, 0.735)	26.5 (25.3, 27.2)	71.2	61.3	63.2	69.5	66.1	1.840	0.470	54.4	38.9
**M**	0.340 (0.300, 0.381)	0.709 (0.684, 0.734)	27.0 (25.8, 27.1)	63.1	69.2	74.6	56.7	65.6	2.049	0.533	49.9	44.7
**WC (cm) W**	0.400 (0.368, 0.435)	0.733 (0.713, 0.754)	89 (85.8, 91.6)	74.6	59.8	63.4	71.6	66.9	1.856	0.425	56.9 ^b^	39.6 ^c^
**M**	0.376 (0.336, 0.418)	0.731 (0.706, 0.756)	93.5 (91.8, 99.8)	76.8	57.5	72.1	63.3	68.8	1.807	0.403	62.7 ^d^	49.2 ^e^
**HC (cm) W**	0.287 (0.249, 0.324)	0.665 (0.643, 0.687)	103.0 (100.0, 108.1)	66.8	56.6	59.0	64.6	61.5	1.539	0.587	54.8	37.7
**M**	0.229 (0.187, 0.275)	0.636 (0.608, 0.663)	99.0 (97.3, 104.2)	71.4	48.4	66.5	54.2	62.0	1.384	0.591	63.4	48.2
**WHtR W**	0.423 (0.389, 0.458)	0.747 (0.727, 0.767)	0.564 (0.549, 0.592)	75.9	61.6	64.9	73.3	68.5	1.977	0.391	56.5	39.9
**M**	0.406 (0.367, 0.444)	0.746 (0.722, 0.770)	0.571 (0.551, 0.584)	66.3	71.6	77.0	59.7	68.5	2.335	0.471	50.9	45.9
**WHR W**	0.303 (0.266, 0.338)	0.678 (0.656, 0.699)	0.844 (0.834, 0.883)	78.1	48.0	58.4	70.1	62.5	1.502	0.456	64.6	41.3
**M**	0.342 (0.299, 0.382)	0.709 (0.684, 0.735)	0.930 (0.922, 0.933)	77.2	58.4	72.6	64.1	69.4	1.856	0.390	62.6	49.6
**BAI W**	0.421 (0.387, 0.456)	0.746 (0.726, 0.766)	27.4 (26.2, 28.5)	74.5	64.3	66.1	72.9	69.2	2.087	0.397	54.5	39.5
**M**	0.402 (0.365, 0439)	0.742 (0.717, 0.766)	25.5 (24.9, 26.5)	70.0	68.5	76.1	61.4	69.4	2.222	0.438	54.3	47.4

W, women; M, men; CMR, cardiometabolic risk; AUC, area under the ROC curve; Sn, sensitivity; Sp, Specificity; PPV, positive predictive value; NPV, negative predictive value; LR, likelihood ratio; Comp., component of the metabolic syndrome; MetS, metabolic syndrome (according to the JIS definition); WC, waist circumference; HC, hip circumference; WHtR, waist-to-height ratio; WHR, waist-to-hip ratio; BAI, body adiposity index; BMI, body mass index.

^a^p<0.001 for all the correlation coefficients

^b^According to the *European* and *Europid* cut-off points for WC the prevalence was 60.2% and 81.0% in women

^c^According to the *European* and *Europid* cut-off points for WC the prevalence was 40.6% and 45.9% in women

^d^According to the *European* and *Europid* cut-off points for WC the prevalence was 32.7% and 62.1% in men

^e^According to the *European* and *Europid* cut-off points for WC the prevalence was 38.8% and 49.0% in men.

WC and WHR showed a good sensitivity for both sexes (74.6% / 78.1% in women and 76.8% / 77.2% in men, respectively); additionally, WHtR and BAI performed well in women (75.9% and 74.5%, respectively). The specificity was low for all cut-off points, especially in women. The WHtR and BAI presented higher and lower positive and negative LR, respectively.

Overall, WC, WHtR, and BAI cut-off points showed a good performance in both sexes, in terms of sensitivity, specificity, predictive value, accuracy and LR.

Using the JIS criteria, and according to the cut-off points tested for all adiposity measures, the prevalence of MetS varied between 37.7% and 41.3% in women and between 44.0% and 49.6% in men. Considering the estimated WC cut-off points, MetS prevalence was 39.6% in women and 49.2% in men. The WC cut-off points determined for this Portuguese sample were closer to the *European* criteria in women and to the *Europid* criteria in men [13].

The prevalence of AO using the estimated WC cut-off points (**≥** 89.0 cm in women and **≥** 93.5 cm in men) was 56.9% in women and 62.7% in men.

## Discussion

In this study, the correlations of WHtR, WC and BAI with elevated cardiometabolic risk were higher than those of other adiposity measures, namely, BMI. These results are supported by other published evidence, which showed that WC and WHtR performed better in detecting CVD and diabetes risk than BMI [3, 11, 12, 22]. In addition, the results are partially explained by the stronger association of those anthropometric measures with VAT [9, 10]. However, BAI performed quite well in our sample, which is not supported by other studies [10, 23, 24].

As expected, differences between genders were observed for the correlations between elevated cardiometabolic risk and the evaluated adiposity measures, as well as for the estimated cut-off points. These differences may be explained by the previously described gender variations in body fat distribution [25, 26]. Compared to women, men have greater total VAT volume and are more likely to accumulate adipose tissue in their upper body [27], whereas women usually accumulate adipose tissue in the lower body. This fact may explain why men show higher VAT values than women for the same WC value [28].

The WHR cut-off points showed a high sensitivity and low specificity in both sexes. However, WHR performed better in men than in women when considering the positive predictive value, accuracy and LR; these observations are supported by previously published studies [29]. The gender differences in body fat distribution could, at least in part, explain the differences in CVD and diabetes risk observed between men and women [30].

According to our data, WHtR was the adiposity measure that best performed in the evaluation of the adiposity component for MetS. As WC is currently used as the adiposity component in the definition of MetS and as no significant differences were observed between this and other measures in our study, it is likely that no modification to the MetS definition need to be proposed. Regarding the good performance of WHtR, this measure may be useful in benchmarking studies of different populations as it allows for differences in Ht [31, 32].

The estimated cut-off points for WC obtained in this study were 89.0 cm in women and 93.5 cm in men. These cut-off points are lower in men than the ones proposed by the National Institute of Health [16]. Therefore, these results suggest that, in the Portuguese population, the *European* cut-off points appear to be more appropriate to prevent over-diagnosis in women. According to the *Europid* cut-offs, the majority of women (81%) met the criteria for the WC component. However, men with a WC of 94.0 cm or more should be carefully assessed.

Recently published results from Spanish studies [33, 34] showed similar cut-off points for WC (88.5 to 89.5 cm in women and 94.5 cm in men), with close WC mean values in men and discretely higher values in women. This similarity with our results may reflect a genetic and epigenetic proximity between the populations. A review, including sixty-one research papers [18], found optimal cut-off values ranging from 65.5 to 101.2 cm for women and 72.5 to 103.0 cm for men; the cut-off points of the European and United States studies were not very different from ours (83 to 88 cm in women and 93 to 96 cm in men). Another review, including studies investigating ethnic-specific WC cut-off points among Aboriginal, Asian, African (SubSaharan), African-American, Hispanic, Middle Eastern, Pacific Islander and South American populations [19], found lower cut-off point in the populations of Asian origin; the evidence was less robust for other ethnic groups.

When comparing the prevalences of MetS, according to the JIS definition, the estimated and proposed (*European* and *Europid*) WC cut-off points showed a closer prevalence between the figure estimated using the *European* cut-off points in women and the *Europid* cut-off points in men.

Furthermore, we found a higher prevalence of MetS in the Portuguese sample than in other South European countries [35–38] and the USA [39]. In addition, the estimated prevalence of AO (based on WC) and overweight was slightly higher in this sample than in previous national studies [40].

Overall, our study was based on cross-sectional data. Prospective studies should be conducted to determine whether the estimated WC cut-off values are more appropriate than the *European* and *Europid* values and, moreover, to estimate the risk of CVD and diabetes in the Portuguese population.

## Conclusions

According to our data, WC, WHtR and BAI are the adiposity measures that provided the best evaluation of the adiposity component for MetS in the Portuguese population.

As WC is currently used as the adiposity measure in the definition of MetS and as no relevant differences were observed between this measure and WHtR, it is likely that no modification to the MetS definition need to be proposed.

The new WC cut-off points proposed for the Portuguese population (89.0 cm in females and 93.5 cm in males) are very similar to the *European* cut-off points in women and the *Europid* values in men.

Use of the *European* cut-off points may be more appropriate in order to prevent over-diagnosis in women.

## Supporting information

S1 Supporting information Data set. (SAV)
